# Translating Antiviral Therapies to Veterinary Use: A Review of Immunomodulatory Agents for Potential Application in Aleutian Mink Diseases

**DOI:** 10.3390/ani15162360

**Published:** 2025-08-11

**Authors:** Marcin Kondracki, Andrzej Żmuda, Magdalena Gryzinska, Ilona Mazurkiewicz, Beata Seremak, Jacek Furmaga, Andrzej Jakubczak

**Affiliations:** 1 Institute of Biological Basis of Animal Production, University of Life Sciences in Lublin, 20-950 Lublin, Poland; marcin.kondracki@up.lublin.pl (M.K.); ilona.mazurkiewicz@up.lublin.pl (I.M.); andrzej.jakubczak@up.lublin.pl (A.J.); 2 Department of Epizootiology and Clinic of Infectious Diseases, University of Life Sciences in Lublin, 20-612 Lublin, Poland; andrzej.zmuda@up.lublin.pl; 3 Department of Animal Reproduction Biotechnology and Environmental Hygiene, West Pomeranian University of Technology, 71-270 Szczecin, Poland; beata.seremak@zut.edu.pl; 4 Department of General and Transplant Surgery and Clinical Nutrition, Medical University of Lublin, 20-954 Lublin, Poland; jacek.furmaga@umlub.pl

**Keywords:** Aleutian mink disease virus, immunomodulation, immunostimulants

## Abstract

Aleutian mink disease virus (AMDV) poses a serious threat to the fur industry worldwide, and the lack of effective treatments or vaccines makes it difficult to combat the disease. There are highly virulent strains of AMDV that cause severe symptoms, but by selecting animals with low titres of anti-AMDV antibodies, it is possible to obtain mink with increased resistance to AMDV. Immunomodulation research offers a promising prospect in combating AMDV. The literature review covered the potential use of immunomodulators, including nanoparticles and macromolecules, which can positively influence the immune response. Previous attempts to treat AMDV have not been satisfactory, nor have attempts to develop a fully effective vaccine. The use of new technologies based on cell engineering and nanotechnologies in the prevention and treatment of diseases has become a fact.

## Simple Summary

Aleutian mink disease virus (AMDV) is a serious threat to the mink due to the lack of effective treatments or vaccines. Due to this fact, minks with low levels of antibodies against AMDV are selected to enable the breeding of more resistant animals. Recent research into immunomodulators, including nanoparticles and organic compounds, shows promise in enhancing immune responses and potentially managing the disease. Immunosuppressants, though typically used to prevent organ rejection, may also alleviate symptoms of viral infections like AMDV. The disease’s severity depends on virus virulence and the mink’s immune system, age, and genetics, often resulting in organ damage, low reproduction rates, and high mortality. Analysis of potential pharmacological treatment of Aleutian Mink Disease (AMD) may open the door to solving this growing problem. The rapid development of technology based on nanomaterials and organic compounds using immunosuppressants with anti-inflammatory properties may in the near future lead to the creation of specific preparations for use in individual treatment.

## 1. Introduction

### 1.1. Aleutian Mink Disease Virus

Aleutian mink disease virus (AMDV) is a virus of the genus *Amdoparvovirus* in the family *Parvoviridae*. The virus poses a serious threat to the health of mink and thus to the fur industry in mink-farming countries [1]. There are many scientific reports regarding its properties and the responses to infection [2]. There is not yet any effective treatment for the disease caused by AMDV, and all attempts at developing an effective vaccine have been unsuccessful. The standard technique for controlling the disease is to eliminate seropositive mink using immunoenzymatic assays such as counterimmunoelectrophoresis (CIEP) or enzyme-linked immunosorbent assay (ELISA) [3].

**Figure 1:**
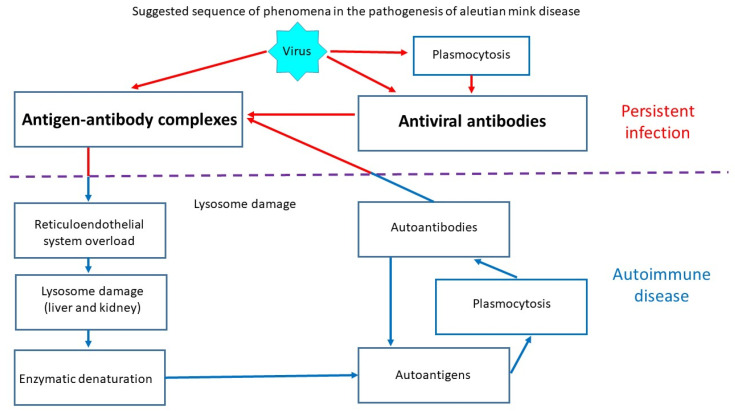
Schematic of Aleutian disease pathogenesis.

The AMDV genotype and its virulence play an important role in the development of Aleutian mink disease (AMD). Highly virulent strains of the virus are particularly dangerous and can lead to high mortality. AMDV infection can lead to acute glomerulonephritis and arteritis as well as progressive disease associated with immune complexes. It can also cause acute interstitial pneumonia, which usually leads to death in neonates. Some animals may exhibit classical symptoms, but the infection is asymptomatic or transient in most non-Aleutian mink (Figure 1). AMD has also been observed in other mustelids, such as otters, martens, ferrets, and badgers [4].

The phenomenon of low antibody levels in healthy but infected mink (non-progressive infection) has long been known. Tolerant. The solution for the moment is immunological monitoring of the company of mink to eliminate individuals with the highest antibody titres.

Tolerant populations of mink can be created by selecting individuals with low antibody levels, as these may also have higher reproductive performance. The combination of a weak positive ELISA result and a normal litter size may help to decrease the incidence of clinical symptoms of the disease. Antibodies also perform preventive functions, as they help to prevent replication of the virus and partially remove and sequester it in the early stages of infection [5].

Identification of the agent inducing disease symptoms is a crucial element in making an accurate and effective diagnosis and planning treatment. Despite attempts to introduce vaccines [6], the most effective solutions remain non-specific prevention, early diagnosis, and control of purchased breeding material [7].

The genome of parvoviruses consists of two open reading frames (ORFs) surrounded by untranslated regions. According to research by Qiu et al. [8], the left ORF encodes nonstructural proteins NS1, NS2, and NS3, while the right ORF codes for capsid proteins VP1 and VP2. These proteins play an important role in the replication of the virus and also regulate transcription of its genetic material [9]. The virus additionally has two structural proteins, VP1 and VP2, in a 1:9 ratio. Among these, VP2 plays an especially important role, as there are great differences in VP2 between strains of the virus, which determine differences in their virulence [10].

However, what distinguishes parvoviruses from other viruses is their extreme plasticity. A change in a single nucleotide in the hypervariable region, and thus in an amino acid, can significantly influence the range of infected hosts, tropism for specific tissues, or virulence. High genetic polymorphism in this region also enables phylogenetic analysis [2,6,7,11].

### 1.2. Immunomodulation (Immunosuppression and Immunostimulation)

The therapeutic revolution of the last two decades in inflammatory immune diseases has led to the use of a variety of drugs, such as immunosuppressants and immunomodulators. This new approach to treatment has resulted in significant progress in rheumatology and has also enabled more effective treatment that is better adjusted to the patient’s needs [12].

Immunomodulators are substances that can be used to target specific antigens or immunogens, which in turn enables modulation of the immune response, thus increasing the therapeutic effects [13]. They are particularly effective as adjuvants of vaccines [14,15,16]. Their specific effect allows the drug to act more selectively and is safer for the patient [17]. Experimental studies on these drugs have been described in numerous scientific publications dealing with diseases of the oral cavity and health [18,19,20], autoimmune diseases [21,22,23], and treatment of transplants and inflammatory bowel disease (APC) [24,25,26,27,28].

β-Glucan is an organic compound from the polysaccharide group. It is a soluble fraction of dietary fibre. The effect of B-glucan is difficult to predict due to the lack of studies on Aleutian mink disease. In studies of autoimmune diseases in humans, its effect on macrophages was determined [29]. A significant reduction in the number of macrophages was found after administration of B-glucan and the lack of exacerbation of disease symptoms. Macrophages also become infected in Aleutian mink disease [30]. B-glucan was supplemented in mink on a fish-based diet, and a reduction in IgG immunoglobulin titres was obtained [31], which, according to the authors, may lead to a reduction in the production of immune complexes and their destructive effects on organs. Hence, the authors’ interest in this preparation.

Drugs, antibodies, and small-molecule inhibitors are included in Table 1. Kowalczyk et al. [32] showed that administration of methisoprinol (isoprinosine), a drug with immunostimulatory properties, to female mink with Aleutian mink disease (AMD) can be effective in combating the disease. The authors found that administration of methisoprinol reduced the viral load of AMDV (the number of copies of DNA per µL) in the spleen and lymph nodes and improved the average body weight of young mink before slaughter. However, the use of isoprinosine, which increases the production of antibodies by B lymphocytes, may exacerbate pathological changes in Aleutian disease due to the increased production of immune complexes deposited in organs. Farid and Smith [33] conducted a study on the effect of seaweed (*Ascophyllum nodosum*) meal on mink infected with AMDV. They found that supplementation with this preparation did not improve the immune response of mink to either infection or replication of the virus, but a lower viral load was observed in the blood of the mink, as well as reduced concentrations of urea, nitrogen (BUN) and creatine, which suggests an improvement in kidney function.

In the case of a disease such as AMDV with a complex pathogenesis, the use of medicinal plants with proven efficacy can yield positive results. According to Ortuño-Sahagún et al. [34], immunomodulators of plant origin, such as turmeric (*Curcuma longa*), sunflower (*Carthamus tinctorius*), and sessile joyweed (*Alternanthera sessilis*), yield promising results, suggesting that these plants can have potential in the treatment of liver disease owing to their immunomodulatory effects. Particularly noteworthy is turmeric, which was effective in treating viral diseases of the liver as a natural immunosuppressant.

In in vitro studies, MSCs significantly inhibit immune cell proliferation and production of pro-inflammatory cytokines and induce apoptosis of T cells [45,46,47,48]. In addition, MSCs reduce the production of B cell antibodies and inhibit the generation and function of antigen-presenting cells [49,50]. Which, according to the authors, could reduce the number of virus-antibody complexes formed and, consequently, lead to a reduction in the severity of destructive changes in organs associated with their deposition. In vivo studies and human clinical samples have shown that systemic MSC transplantation is effective in treating various autoimmune diseases, such as graft-versus-host disease (GVHD), systemic lupus erythematosus (SLE), rheumatoid arthritis (RA), and Crohn’s disease (IBD) [47,48,70]. MSCs regulate the local immune environment and are a suitable ‘substrate’ for tissue regeneration [71,72,73,74]. Many therapeutic mechanisms contribute to MSC-based cellular treatment, including paracrine secretion and interactions between MSCs and immune cells [74,75,76,77].

### 1.3. Immunosuppression Mechanisms

Immunosuppression is the process of reducing the ability of the immune system to effectively respond to foreign antigens, including those located on the surface of the body’s own cells, and to pathogens. In some cases, this may be the effect of destruction of immune effector cells or blocking of intracellular signalling pathways, which are essential to the recognition of antigens and activation of the immune response [78].

Synthetic drugs and biologically active substances are often used in immunosuppressive therapy. Cyclophosphamide, an active immunosuppressive agent, was used in one of the first attempts to pharmacologically alleviate the symptoms of Aleutian Mink Disease (AMD) by reducing hypergammaglobulinemia and organ lesions. However, the drug was not tolerated well by the mink at the dose used [52]. Their long-term use may cause systemic toxicity or immune deficiency, which can result in further health problems [53].

Studies using mycophenolate and calcineurin inhibitors have confirmed the effectiveness of immunosuppressive therapy [79]. Wojciechowski et al. [80], in a study on the effect of maintenance immunosuppression on transplants, found that it can have short-term benefits, but that long-term use can have negative effects.

In developing a therapeutic strategy for reducing immunity, both the innate and adaptive immune responses must be taken into account. Innate immunity consists of the initial activation of neutrophils and macrophages, which then release cytokines and induce local inflammation at the site of the reaction [53,81,82]. The innate immune response is followed by an adaptive immune response specific to a given antigen, which involves activation of T and B cells. The effectiveness of an immunosuppressive drug depends on which metabolic pathway of the immune system it affects, as the innate and adaptive responses are linked [81,83,84].

New technologies are increasingly being used successfully in the treatment of some human and animal diseases. Hence, attempts can also be made to use at least some of them in the treatment of Aleutian disease. Studies have recently been conducted on the exploitation of the immune response to nanoparticles as new drugs with direct immunostimulatory or immunosuppressive effects, despite the fact that the body’s basic reactions to nanomaterials and their structure and activity are not fully known [85]. Many research teams are searching for new immunostimulants, such as nanoparticle-supported or nanoparticle-based vaccines [86,87]. Ngobili and Daniele [53] showed that it is possible to treat autoimmune diseases, mitigate allergic reactions, and avoid transplant rejections through inhibition of immune signalling pathways by nanoparticles of metal or metal oxides (e.g., iron oxide), carbon nanomaterials, polymer nanoparticles, or macromolecules. Important metal nanoparticles include gold and silver nanoparticles from 5 to 35 nm in size, and also polyacrylate-coated gold nanoparticles 200 nm in diameter. The authors used nanoparticles of varying types and sizes and pointed out differences in immunosuppressive interactions with cells of the innate immune response, including macrophages, dendritic cells, neutrophils, mast cells, and NK cells, as well as the adaptive immune response (T and B cells).

Low-molecular-weight drugs such as azathioprine, sirolimus, and everolimus, as well as inhibitors of nucleotide synthesis, including various forms of mycophenolic acid and calcineurin inhibitors, e.g., tacrolimus and cyclosporine, can be useful in immunosuppressive therapies [58,59]. Agonists of the glucocorticoid receptor, on the other hand, act by attaching to and activating it, modifying the production of transcription factors such as activator protein 1 and nuclear factor-κB, and then modifying the properties of the cell. These preparations can increase the production of cytokines, which stimulate cell division and affect nearby cells.

Samuel and Kimmoun [64] showed that immunosuppressive drugs, including corticosteroids, can promote replication of the hepatitis B virus (HBV). This takes place through direct and indirect mechanisms reacting to glucocorticoids in the HBV genome, which leads to increased gene expression [88]. The risk of reactivation of HBV is associated with the degree and duration of immunosuppression and various risk factors, including specific viral markers and certain high-risk treatments, such as rituximab and TNF-α inhibitors [89]. Monitoring of patients with present or past HBV infection under immunosuppressive conditions is crucial, and initiation of antiviral treatment based on immunosuppressive drugs may be helpful in treatment [89].

A potential side effect of immunosuppression is the development of occult tumours or metastatic cancer cells in the case of organ or tissue transplants [90]. Loren et al. [91] showed that interrupting immunosuppressive drug therapy can cause regression of transplanted tumours together with the organs or tissues.

Immunosuppressive drugs can be used to control immune reactions, particularly in autoimmune diseases and in the case of transplantations, affecting the entire immune system as well as those lymphocytes that are responsible for damage to or rejection of transplants. Targeting immunosuppression at specific cells without disturbing the rest of the immune system is a better solution. Waldmann [92] reported that research in mice showed that short-term treatment using monoclonal antibodies against specific lymphocyte molecules can lead to the induction of tolerance.

## 2. Immunosuppressive Preparations

Corticosteroids, also known as glucocorticoids, are a class of steroid hormones produced by the adrenal cortex, which have been used as immunosuppressive drugs for many years. They have pleiotropic effects, which means that they act on multiple systems and target various types of cells, and their effects are different at different doses [56]. At low doses, corticosteroids act through an intracellular glucocorticoid receptor (GR), regulating gene expression. GR binds to specific genes and interacts with transcription factors such as the NF-kb and AP-1 complexes, inhibiting synthesis of pro-inflammatory cytokines and adhesion proteins [93]. At high doses, however, corticosteroids act through non-genomic mechanisms, such as changes in cell membrane viscosity and in levels of intracellular calcium, as well as by interacting with signalling pathways, e.g., MAPK (mitogen-activated protein kinase) signalling pathways. High doses can also lead to regulated apoptosis in thymocytes [65]. It is important to test the mechanisms of action of corticosteroids used in treatment or combined with other immunosuppressive drugs in clinical practice. The potential side effects of corticosteroids should also be considered in choosing a treatment regime [65,66,67,68].

Cyclosporine, a new-generation immunosuppressive drug, specifically and reversibly inhibits T cells. It has been used for many years in diseases requiring transplants due to its ability to block the graft-versus-host reaction. Other indications include atopic dermatitis, chronic idiopathic urticaria, lichen planus, pyoderma gangrenosum, alopecia areata, granuloma annulare, and several other skin diseases [69]. Another type of substance used in immunosuppression, i.e., suppression of the body’s immune response, is cyclophilins. These are proteins with the ability to bind immunosuppressive drugs such as cyclosporine A (CsA), tacrolimus (FK506), and rapamycin [63]. Naoumov [42] and Hopkins [43] showed that cyclophilins CypA, CypB and CypD may also be involved in the replication of some subgroups of RNA viruses. According to de Wilde et al. [44], cyclophilin A (CypA) is a protein which plays an important role in the replication of RNA viruses, particularly coronaviruses and influenza viruses, by inhibiting their replication.

mTOR inhibitors (such as rapamycin and everolimus) are used in treatment to prevent the rejection of solid organ transplants. Rapamycin, produced by *Streptomyces hygroscopicus*, is a macrolide antibiotic; everolimus is its analogue with a shorter half-life. Both of these drugs bind to the protein kinase mTOR, which regulates cell growth and proliferation and protein synthesis [61]. mTOR inhibitors block mainly TORC1 and thereby inhibit the cell cycle from the G1 phase to the S phase [62]. They also influence the PI3/Akt/mTOR signalling cascade, increasing cell proliferation mediated by various cytokines and growth factors, such as IL-2/receptor IL-2, IL-11, G-CSF, IGF-1, VEGF, EGF, and erythropoietin. mTOR inhibitors do not exhibit nephrotoxicity but can delay convalescence following tubular necrosis and increase nephrotoxicity associated with calcineurin inhibitors (CNI). The direct effect of mTOR inhibitors on the podocytes can lead to damage to the integrity of the vascular wall of the glomerulus and cause proteinuria [60]. Clinical studies suggest that mTOR inhibitors reduce the risk of cancer following organ transplant, which may be linked to inhibition of the mTOR pathway (Akt/PI3K) and blocking of mRNA translation of some procarcinogenic factors, such as VEGF, cyclin D1, and IL-10 [51]. mTOR inhibitors are metabolised by cytochrome P450 enzymes (CYP3A4), and their concentrations may be affected by P-glycoprotein, which can result in drug interactions, as in the case of calcineurin inhibitors [56].

Immunosuppressants, such as calcineurin inhibitors (CNI), cyclosporines (CyA), and tacrolimus (TAC), are commonly used to treat autoimmune diseases and transplants. However, recent studies suggest that these drugs can also affect the replication of coronaviruses [94]. In vitro and in vivo studies have shown that CyA and TAC bind to various cellular cyclophilins and inhibit the effects of calcineurin, which affects the transcription of genes coding for key cytokines taking part in immune mechanisms. Moreover, cyclophilins are necessary for the replication of viruses. These discoveries suggest that patients treated with immunosuppressants may be more susceptible to coronavirus infection [61,95]. Cyclophilin inhibition by CyA has been shown to effectively block replication of CoV of all types, including SARS-CoV, human CoV-229E and NL-63, feline CoV, and infectious bronchitis virus (IBV) in birds. This is an interesting discovery, because the ability of CyA to inhibit cyclophilins could potentially provide broad-spectrum solutions for the treatment of various CoV infections [62].

Mycophenolic acid has been described as a potential inhibitor of the replication of MERS-CoV. Research has shown that it can block the effects of papain-like protease, a key enzyme for replication of the virus, in vitro [60].

Azathioprine, as a purine analogue, exerts its effect by inhibiting synthesis of nucleic acids. Azathioprine, by weakening the body’s immune response, could contribute to reducing humoral immunity against the AMD virus, which would reduce the number of immune complexes formed. However, an adverse effect of azathioprine use would be increased susceptibility of farmed minks to other infectious diseases such as salmonellosis. Following metabolism in the liver, it is converted to powerful compounds, thioinosinic acid and 6-methylmercaptopurine. The key driver of the effect of azathioprine is thioinosinic acid, a guanine analogue which disrupts RNA and DNA synthesis, leading to a cytotoxic effect on white blood cells. The breakdown of the by-products of azathioprine is regulated by the enzyme xanthine oxidase [51]. Mycophenolic acid (MPA) is the main component of two pharmaceutical drugs: mofetil mycophenolate (MMF) and enteric-coated sodium mycophenolate (MPS). This substance acts as a reversible and non-competitive inhibitor of the enzyme inosine monophosphate dehydrogenase (IMPDH), responsible for the conversion of inosine monophosphate to guanine. Inhibition of this enzyme reduces the capacity for guanine production and DNA replication. MPA is directed at both isomers of IMPDH, constitutive and inducible, with a stronger effect on the latter, which is expressed during cellular activation. In consequence, T and B cells, which rely on the de novo purine synthesis pathway, are the main cells affected by MPA. In addition, this drug reduces the transfer of fucose and mannose residues from glycoproteins, impeding production of integrin VLA-4 and reducing adhesion of leukocytes to vascular cells [56,57].

Nanoparticles play an important role in medicine because their size, shape, and chemical properties make it easier for them to bind with elements of the circulatory and immune systems, which translates to more effective interaction with the body and a more effective immune response. The physicochemical properties of nanoparticles are decisive for their interactions with cells, and gold nanoparticles are an excellent example of this type of interaction [96]. Bregoli et al. [35] investigated the interaction of nanoparticles with bone marrow stem cells and K562 and HL-60 cells. They characterised nanoparticles of gold, silver, iron, antimonate and titanium using photon correlation spectroscopy. The physicochemical properties of nanoparticles facilitate interactions with cells and the immune response. Gold nanoparticles have been cited as an example of systems illustrating these effects [35].

Nanotoxicity remains widely unknown, despite the growing number of products containing nanoparticles (NPs) [36]. NPs are obtained not only from nanotechnology but also as by-products of various forms of thermal degradation, such as combustion engines, power plants, combustion plants, and metallurgy. Routes of exposure to NPs include inhalation, accidental or intentional ingestion, absorption through the skin, and injection [37]. Studies in humans show that NPs can enter the bloodstream within one minute after being inhaled [38] and may be present in internal organs such as the liver, kidneys, and intestines [35,39,40,41].

Nanoparticles of metal oxides, including oxides of iron and precious metals, such as gold and silver, exhibit immunosuppressive and anti-inflammatory activity. Studies have shown that iron oxide nanoparticles reduce the immune response, including the humoral response, which involves recognition of antigens by B cells. Injection with organic gold compounds has been used to treat inflammation for several decades, and recent reports provide new information regarding the biochemical pathway by which gold nanoparticles affect the inflammatory state [97]. Citrate-coated gold nanoparticles are harmless for cells and organs and exhibit anti-inflammatory activity, inhibiting cellular responses. Monodisperse citrate-coated gold nanoparticles of various sizes have been assessed in terms of their effects on pro-inflammatory functions, including production of interleukin 1 beta (IL-1β) [53,96].

Silver nanoparticles are used less often than gold nanoparticles in studies on immunosuppression. However, studies suggest that silver nanoparticles influence the production of cytokines, which play an important role in wound inflammation. The use of silver nanoparticles, both locally and systemically, reduces inflammation and levels of pro-inflammatory cytokines. Further research on the interactions of silver nanoparticles with the immune system may contribute to the development of new applications in areas such as medicine, antimicrobial systems, and drug production [53,97].

Nanomaterials are among the most modern solutions in medicine. Among these we can distinguish carbon nanomaterials, which are used successfully as anti-inflammatory agents owing to their strong free radical scavenging properties [98]. Fullerenes have also proven effective at reducing the level of oxygen free radicals, owing to their effective reaction with them [99]. Moreover, fullerenes block certain properties of radicals, reducing reactive oxygen species of both hydroxyl and superoxide radicals. In vitro and in vivo studies have demonstrated that fullerenes effectively suppress oxidative stress; however, the immune response is dependent on the dosage and delivery [100].

Polymeric nanoparticles and macromolecules are an alternative to inorganic and carbon nanomaterials. Nanomaterials from polymers and other macromolecules are often used to induce immunostimulatory reactions. Most polymeric nanoparticles and polymer macromolecules are also used as drug carriers to induce indirect immunosuppression [53]. As nanoparticles of heavy metals are harmful, they are not used to induce immunosuppression despite their immunotoxic properties.

Alexandersen et al. [101] showed that the administration of polyclonal anti-IgM antibody resulted in effective inhibition of B lymphocytes and production of specific anti-AMDV antibodies. This treatment resulted in mink pups not developing the respiratory failure typical of an acute form of Aleutian disease. Suppression of B lymphocytes resulted in increased replication of AMDV, which resulted in the presence of inclusion bodies in type II pneumocytes in these animals. Induction of effective immunosuppression with anti-IgM led to complete suppression of anti-AMDV and other IgM antibody production. As the specific anti-AMDV antibodies do not have virus-neutralising properties but contribute to the formation of immune complexes that damage organs, blocking their production could potentially be used to treat Aleutian mink disease. Monoclonal antibodies, and in humans humanised monoclonal antibodies, prevent the proliferation of activated T lymphocytes, binding to Tac-antigen (monoclonal receptor for T cell growth factor). As they have no negative effect on resting T cells, they do not impair immunity [102]. Mouse monoclonal anti-CD3 antibodies, chimeric antibodies, humanised anti-CD25 antibodies, and humanised anti-CD52 antibodies are commercially available [103]. The latest generations of monoclonal antibodies inhibit activation of cytokines and T and B cell-dependent costimulation, block activation of complement, and also destroy immune cells responsible for transplant rejection [55]. Chimeric monoclonal antibodies against TNF-α and IL-6 have significantly improved the treatment of rheumatoid arthritis. Drugs based on them bind membrane-bound and soluble TNF-α, which is mainly responsible for the inflammatory process [54].

Certain monoclonal antibodies, including anti-IgM, have shown potential for selective immunosuppression in mink models by reducing the production of non-neutralising antibodies that form pathogenic immune complexes in Aleutian disease. Although studies on transplantation tolerance using anti-CD4 and anti-CD8 antibodies have been conducted in mice, such models have limited applicability in mink due to a lack of data on MHC compatibility or transplant immunology in this species. Therefore, future studies should prioritise direct evaluation of monoclonal antibodies targeting B or T cells in mink, assessing their ability to modulate immune response without increasing susceptibility to secondary infections [92,101].

## 3. Discussion

Immunosuppressive strategies, although originally developed in human medicine primarily for transplant tolerance, have also been explored in veterinary contexts as supportive therapy in chronic viral infections such as Aleutian mink disease virus (AMDV). In mustelids, including mink and ferrets, the immune system exhibits species-specific responses, notably a heightened histamine release, which may contribute to exaggerated inflammatory processes. The domestic ferret (*Mustela putorius furo*), closely related to the American mink, has been used as an experimental model for studying the pathogenesis and immune response during Aleutian mink disease virus (AMDV) infection. In ferrets, limited activation of B lymphocytes and lower levels of immune complexes have been observed, suggesting a distinct pattern of humoral immune response activation compared to mink [104]. Immunological studies have shown that AMDV induces changes in type I interferon expression and signalling pathways associated with Toll-like receptors in ferrets, which may influence the early antiviral immune response. Due to their relative ease of handling, suitability for repeat procedures, and the availability of advanced immunological tools, ferrets represent a valuable experimental model for evaluating immunomodulators and potential vaccines against AMDV. A study by Blank et al. [105] demonstrated that approximately half of domestic ferrets tested had antibodies against AMDV, although none showed detectable viral DNA. The authors suggest this may be due to the virus being in a latent phase or samples being taken during a non-viraemic state [105]. Furthermore, evidence suggesting that AMDV is either non-pathogenic or only mildly pathogenic in ferrets and polecats stems from the observation that these animals do not exhibit elevated gamma-globulin levels, a common finding in AMDV-infected mink [106].

Progress in AMDV diagnostics in recent years has focused on improving molecular tests, primarily PCR-based (polymerase chain reaction) methods, which enable the detection of the virus’s genetic material even at a very early stage of infection, before antibodies appear. Methods such as real-time qPCR, droplet digital PCR (ddPCR), and LAMP (loop-mediated isothermal amplification) have increased the sensitivity and specificity of AMDV detection in blood, spleen, lymph node, and even environmental samples (e.g., bedding and air in animal husbandry facilities) [107,108,109]. At the same time, serological tests—such as ELISA and counter-immunoelectrophoresis (CIEP)—remain extremely important tools for assessing the immune response and detecting chronic infections. In population screening, serological tests detect animals that have been exposed to the virus, which is especially important in cases of low-level or latent infections. However, it should be noted that serological tests are subject to a window period (up to several weeks) and do not detect infected animals when the body is just beginning to produce antibodies (after infection) and their levels are still too low for the test to detect them. As a result, the test may yield a negative result even though the animal is infected. Currently, complementary use of both approaches is recommended: molecular tests are useful in identifying active infection and monitoring viral status (especially in early-infected or asymptomatic individuals), while serological tests allow for the assessment of the stage of infection and the strength of the humoral response. This two-pronged approach increases diagnostic accuracy and allows for better management of animal selection for immunity or tolerance [7].

Unlike in humans, therapeutic immunomodulation in mink must consider colony-level application, cost-effectiveness, and practical feasibility. Studies in mink have shown that certain immunosuppressive interventions, such as anti-IgM antibodies, can modulate humoral responses and reduce the formation of pathogenic immune complexes in AMDV infection. While novel immunological tools such as monoclonal antibodies and targeted biologics are advancing rapidly in human medicine, their application in mink is currently limited by the lack of species-specific reagents and the high cost of development. Therefore, future efforts should focus on better characterisation of the mink immune system and pharmacological evaluation of existing immunomodulators in this species. An alternative and potentially more feasible approach may involve the development of effective vaccination strategies, including those based on mRNA platforms.

AMDV is a single-stranded DNA virus with a small genome of about 4.8 kb. It is a parvovirus with two open reading frame sequences (ORFs) crucial to its functioning and replication [104]. The most serious form of the disease permanently affects adult animals. Its onset involves the immune system, which causes an excessive increase in plasma cells, inadequate levels of gamma globulin in the blood, the formation of infectious immune complexes, and nephritis [110]. Owing to immunosuppression, toxic immune complexes are present in smaller quantities, resulting in less organ damage that occurs more slowly.

The course of Aleutian mink disease may vary depending on the virulence of the strain of infecting virus, the age of the mink, the state of the immune system, and the animal’s genotype. T and B cells caused to proliferate by the specific antigen provide optimal conditions for rapid replication of AMDV, leading to immune system dysfunction and impaired immunity. This causes pathological changes in organs, resulting in numerous deaths and low profitability for the breeder, associated with the low average litter size. Analysis of potential pharmacological treatment of Aleutian Mink Disease (AMD) may open the door to solving this growing problem.

The rapid development of technology based on nanomaterials and organic compounds using immunosuppressants with anti-inflammatory properties may in the near future lead to the creation of specific preparations for use in individual treatment.

In formulating strategies to reduce the immune response, both the innate and acquired immune responses should be taken into account. Innate immunity involves activation of neutrophils and macrophages as the first line of defence, which releases certain cytokines causing local inflammation at the site of the reaction. This inflammation is usually followed by an adaptive immune response, which activates antigen-specific cells, T cells, and B cells in order to identify the pathogen or other initiator of the immune response. The innate and adaptive responses are intertwined, and sensitivity to an immunosuppressive drug largely depends on which immune pathway is threatened. Research takes into account suppression of both innate and acquired immune responses, with particular focus on reducing the inflammatory response using nanoparticles (anti-inflammatory activity) [53].

Targeted therapies for inflammatory immune diseases were developed in the last two decades, mainly for rheumatic conditions. Targeted treatments involve blocking of tumour necrosis factor (TNF), receptors of interleukin (IL)-6, IL-1, IL-17, and IL-12/23, B cell depletion, and costimulation. Type I interferons and granulocyte macrophage colony-stimulating factor (GM-CSF) are two more targets which continue to be a subject of research. Inhibition of Janus kinase (JAK) has recently offered treatments adjusted to individual needs. These drugs have proven revolutionary, as they offer unprecedented effectiveness at relatively low risk. The risk of development of a serious infection while taking a TNF inhibitor is marginally higher than in the case of a traditional synthetic drug modifying the disease course, but notably lower than in the case of drugs such as high-dose glucocorticoids [12].

Immunosuppressants and immunomodulators are pharmacologically distinct classes of drugs, differing in their mechanisms of action and impact on the immune system. In veterinary medicine, immunomodulatory treatments have been explored in diseases involving immune complex formation, such as caprine arthritis encephalitis (CAE) in goats and immune complex-mediated synovitis in poultry. For example, the use of immunosuppressants such as azathioprine or cyclophosphamide has been experimentally investigated to modulate chronic inflammatory responses in CAE [111]. Similarly, corticosteroids and immunomodulatory strategies have been tested in broiler chickens with immune complex-induced arthritis to evaluate their impact on inflammation and joint pathology [112]. These veterinary models are more applicable to the pathogenesis of Aleutian disease in mink, which also involves immune complex deposition and chronic immune activation.

In comparing targeted therapies with immunosuppressive drugs, it should be noted that the immune system is a complex system that protects the body against foreign ‘invaders’ using a ‘belt and braces’ strategy. Therefore, once the allograft is discovered, several effector mechanisms of resistance are activated synchronically in order to quickly destroy the ‘invader’. In effect, to reverse the rejection of the allograft, many effector mechanisms of resistance must be switched off, which inevitably increases the risk of infection. On the other hand, IMIDs (immune-mediated inflammatory diseases) are complicated disorders in which many small inherited defects in regulatory immune pathways work together with environmental stimuli, causing chronic inflammation and the development of autoreactivity [12].

## 4. Conclusions

Over the years, many animal viruses that have had destructive effects on health and the economy have appeared in many parts of the world. The Aleutian mink disease virus (AMDV), the most serious disease in mink breeding, is the best example.

In the future, to prevent severe losses caused by AMDV, the development of effective treatment methods and, especially, the development of an effective vaccine are crucial.

Depending on the level of effectiveness of the preparations used, it is possible to analyse the financial impact of the action taken. Breeders aim to have AMD-free animals with their actions, but when this disease occurs, it is necessary to minimise losses and obtain satisfactory breeding results (average young reared). In mink, on which immunological selection is not carried out, the average number of young reared drops to a level of 2.5–3 young per female. In contrast, by selecting animals on the basis of various criteria (e.g., ELISA), it is possible to obtain an average of more than 4.5 young per female, which significantly modifies the financial result. This requires further detailed research.

The development of measures to alleviate the effects of the disease is justified. The disease cannot be treated due to the lack of a fully effective pharmacological treatment. Disinfection of the premises (cages) is not effective. The solution for the moment is immunological monitoring of the company of mink to eliminate individuals with the highest antibody titres.

## Figures and Tables

**Table 1:** List of candidate drugs, antibodies, and small molecule inhibitors against AMDV.

Substance/Preparation	Active Substance	Group	Results of Using the Drug Against AMDV *	References
Turmeric (*Curcuma longa*)	Curcumin	Anti-inflammatory	+	[34]
Methisoprinol (Isoprinozin)	Inosine pranobex	Antiviral	+	[32]
Nanoparticles (Gold, Silver, Iron oxide)	Gold, Silver, Iron oxide	Broad-spectrum immunomodulators	+/−	[35,36,37,38,39,40,41]
Cyclophilins (CypA, CypB, CypD) Mesenchymal stem cells (MSCs)	Cyclophilin A, B, D	Immunomodulators	+	[42,43,44]
	Stem cells		+	[45,46,47,48,49,50]
β-glucans Seaweed meal (*Ascophyllum nodosum*)	β-glucans	Immunostimulants	+	[30,31]
	Fucose, Alginates, Polyphenols		+/−	[33]
Azathioprine	Azathioprine	Immunosuppressive	−	[51]
Cyclophosphamide	Cyclophosphamide		−	[52,53]
Cyclosporine A (CsA)	Cyclosporine A		+	[42,43,44]
IL-6 inhibitors (e.g., Tocilizumab)	Tocilizumab		+	[54]
Janus kinase (JAK) inhibitors	Tofacitinib, Baricitinib		+	[12]
Monoclonal antibodies (Anti-CD25, Anti-CD52)	Basiliximab, Alemtuzumab		+	[54,55]
Monoclonal antibodies against CD3, CD25, CD52	Muromonab, Basiliximab, Alemtuzumab		+	[54,55]
Mycophenolate mofetil (MMF)	Mycophenolate mofetil		+/−	[56,57]
Tacrolimus (FK506)	Tacrolimus		−	[58,59]
Thiopurine analogues	Thioguanine, Mercaptopurine		+/−	[60]
Everolimus (RAD001)	Everolimus	Immunosuppressive, mTOR inhibitors	+	[58,59]
Inhibitors of mTOR (e.g., Everolimus, Rapamycin)	Everolimus, Rapamycin		+/−	[51,60,61,62]
Rapamycin (Sirolimus)	Sirolimus		+	[61,63]
Corticosteroids (Glucocorticoids)	Hydrocortisone, Prednisone	Steroids	+	[56,64,65,66,67,68,69]

* +/− positive/negative.

## Data Availability

All data are included in the manuscript; however, if further information regarding data availability is needed, the corresponding author will provide it upon special request.

## Abbreviations

The following abbreviations are used in this manuscript:

AMDV	Aleutian mink disease virus
AMD	Aleutian mink disease
CIEP	Counter immunoelectrophoresis
ELISA	Enzyme-linked immunosorbent assay
ORF	Open reading frames
MSCs	Mesenchymal stem cel
GVHD	Graft versus host disease
SLE	Systemic lupus erythematosus
RA	Rheumatoid arthritis
IBD	Crohn’s disease
HBV	Hepatitis B virus
TNF	Tumour necrosis factor
GR	Glucocorticoid receptor
MAPK	Mitogen activated protein kinase
NF-κB	Nuclear factor kappa-light-chain-enhancer of activated B cells
CsA	Cyclosporine A
CNIs	Calcineurin inhibitors
TAC	Tacrolimus
SARS-CoV-2	Severe acute respiratory syndrome coronavirus
MERS-CoV	Middle east respiratory syndrome coronavirus
MMF	Mofetil mycophenolate
MPS	Enteric-coated sodium mycophenolate
IMPDH	Inosine monophosphate dehydrogenase
NPs	Nanoparticles
GM-CSF	Granulocyte macrophage colony-stimulating factor
JAK	Janus kinase inhibitor
RMD	Rheumatic and musculoskeletal diseases
ACR	American College of Rheumatology
AZA	Azathioprine
IMIDs	Immune-mediated inflammatory diseases
NHL	Non-Hodgkin’s lymphoma
AIDS	Acquired immunodeficiency syndrome
